# Prevalence, Antimicrobial Resistance and Toxin-Encoding Genes of *Clostridioides difficile* from Environmental Sources Contaminated by Feces

**DOI:** 10.3390/antibiotics12010162

**Published:** 2023-01-12

**Authors:** Khald Blau, Claudia Gallert

**Affiliations:** Department of Microbiology–Biotechnology, Faculty of Technology, University of Applied Sciences Emden/Leer, 26723 Emden, Germany

**Keywords:** *Clostridioides difficile*, antimicrobials, resistance, toxin-encoding genes, qPCR, feces

## Abstract

*Clostridioides difficile* (*C. difficile*) is the most common pathogen causing antibiotic-associated intestinal diseases in humans and some animal species, but it can also be present in various environments outside hospitals. Thus, the objective of this study was to investigate the presence and the characteristics of toxin-encoding genes and antimicrobial resistance of *C. difficile* isolates from different environmental sources. *C. difficile* was found in 32 out of 81 samples (39.50%) after selective enrichment of spore-forming bacteria and in 45 samples (55.56%) using a TaqMan-based qPCR assay. A total of 169 *C. difficile* isolates were recovered from those 32 *C. difficile*-positive environmental samples. The majority of environmental *C. difficile* isolates were toxigenic, with many (88.75%) positive for *tcdA* and *tcdB*. Seventy-four isolates (43.78%) were positive for binary toxins, *cdtA* and *cdtB*, and 19 isolates were non-toxigenic. All the environmental *C. difficile* isolates were susceptible to vancomycin and metronidazole, and most isolates were resistant to ciprofloxacin (66.86%) and clindamycin (46.15%), followed by moxifloxacin (13.02%) and tetracycline (4.73%). Seventy-five isolates (44.38%) showed resistance to at least two of the tested antimicrobials. *C. difficile* strains are commonly present in various environmental sources contaminated by feces and could be a potential source of community-associated *C. difficile* infections.

## 1. Introduction

*Clostridioides* (*Clostridium*) *difficile* is an obligate anaerobic, spore-forming, Gram-positive rod-shaped, and toxin-producing bacterium. *C. difficile* is among the most common nosocomial pathogens that cause antibiotic-associated diarrhea and pseudomembranous colitis worldwide [1,2,3]. The occurrence of *C. difficile* was well documented in hospitalized patients with *C. difficile* infection (CDI) but is also emerging in various environmental sources outside healthcare institutions. Little is known about environmental *C. difficile* isolates, and few studies were conducted on the prevalence, antimicrobial resistance, and toxin-encoding genes of environmental *C. difficile* in environmental sources contaminated with feces (e.g., biogas plants, digested sludge-amended soil, soil, animal feces, manure and in wastewater, raw sludge, and anaerobically digested sewage sludge). The ecology of *C. difficile* outside clinical settings is not fully understood, but the evolution of environmental pathogenic strains could occur in their zoonotic and environmental reservoirs. Therefore, optimization methods for the isolation and detection of *C. difficile* are required to elucidate the role of non-clinical sources as transmission routes of human infection.

*C. difficile* has several virulence factors, including toxins A and B, which are encoded by *tcdA* and *tcdB* genes, respectively, that are localized on a 19 kb Pathogenicity Locus (PaLoc) [4]. In addition, the *C. difficile* toxin CDT (*cdtA* and *cdtB*), which belongs to a family of binary toxins, was identified in toxigenic *C. difficile* strains [5].

However, antimicrobial treatment plays an important role in the development of CDI. *C. difficile* is resistant to many of the antimicrobial agents that are used in humans and animals and can colonize an uncontested niche in the intestine in the presence of antimicrobials that are not active against *C. difficile*, disrupting the natural gut microbiota and favoring the proliferation of *C. difficile* [1,2,6,7]. Although nearly all classes of antimicrobial agents were associated with the risk of CDI such as clindamycin, third-generation cephalosporins, penicillins, and fluoroquinolones continue to be associated with the highest risk for CDI [6,7,8,9,10,11,12]. The usual antimicrobial treatment for CDI requires the use of antimicrobial agents with activities against *C. difficile*, including metronidazole, vancomycin, and fidaxomicin. Hence, they are effective in the treatment of primary and recurrent CDI [7,10]. CDI treatment is complex because of the intrinsic *C. difficile* that acquired multi-resistance to antimicrobials. The resistance to antimicrobial agents is regularly surveyed for human and animal *C. difficile* isolates [9,13,14,15], but antimicrobial resistance in environmental isolates is scarce. Antimicrobial resistance is assumed to play a crucial role in the infection by disseminating *C. difficile*.

Recently, the occurrence of *C. difficile* was reported in different environments: farm cattle animals [9,16], companion animals [17], home garden environments [18], puddle water and soil [19,20], composts and animal manure [21], broiler feces, poultry manure, fertilized soil with poultry manure and dust [22], and wastewater treatment plants (WWTPs) [20,23]. The prevalence of *C. difficile* in these environments may play an important role in disseminating toxigenic *C. difficile* strains into the agricultural environment, which can serve as a potential source of community-associated *C. difficile* infection.

Toxigenic *C. difficile* strains in fecal samples are traditionally detected by culture-dependent methods using selective media or by their toxins via enzyme immunoassay (EIAs). The use of culture-independent methods, such as real-time quantitative PCR (qPCR), has been widely used to detect and quantify the 16S rRNA-specific gene for *C. difficile* [24,25,26,27], toxin-encoding genes (*tcdA* and *tcdB*) of *C. difficile* [27,28], and the *C. difficile* Chaperonin-60 (*cpn60*) gene [29] in fecal samples.

The objectives of this study were to isolate and characterize environmental *C. difficile* strains from different environmental sources contaminated by feces. Toxin-encoding genes and antimicrobial resistance patterns of environmental *C. difficile* isolates were analyzed. In addition, the detection and quantification of environmental *C. difficile* in fecal environmental samples by 16S rRNA gene TaqMan real-time quantitative qPCR assay was determined and compared with the results of the *C. difficile* selective enrichment culture.

## 2. Results

### 2.1. Prevalence and Isolation of C. difficile from Fecally Contaminated Environmental Samples

The environmental *C. difficile* strains were isolated from 32 out of 81 (39.50%) fecally contaminated environmental samples (feces of calves (*n* = 10), biogas plant (*n* = 2), soil (*n* = 1), WWTP samples (*n* = 12), digested sludge-amended soils (*n* = 3), thermophilic digesters of biowaste or sewage sludge (*n* = 2), and anaerobic lab-scale bioreactors for the thermophilic digestion of sewage sludge (*n* = 2)), after the selective enrichment culture from spores in a *C. difficile* selective broth, supplemented with 0.1% sodium taurocholate for spore germination, 16 mg/L norfloxacin, and 32 mg/L moxalactam. The results of the presence of *C. difficile* in different fecal environmental samples are summarized in Table 1. Most *C. difficile* strains were isolated from digested sludge-amended soils and biogas plant samples, followed by WWTP samples, samples from the thermophilic digesters of biowaste or sewage sludge, soil, and the feces of calves at 100%, 75%, 66.67%, 50%, and 31.25%, respectively. However, *C. difficile* was not at all detected in adult cow feces, mixed storage cattle manure, treated sewage (effluent), grass and maize silages, and horse feces. A total of 169 environmental *C. difficile* isolates (WWTP samples (*n* = 69), calf feces (*n* = 40), digested sludge-amended soils (*n* = 21), anaerobic lab-scale bioreactors for the thermophilic digestion of sewage sludge (*n* = 17), thermophilic digesters (*n* = 16), biogas plant (*n* = 5), and soil (*n* = 1)) were isolated from those 32 *C. difficile*-positive samples, after purification by re-streaking them on an appropriate media, as described in Materials and Methods. Then, the isolates were confirmed as *C. difficile* via a latex agglutination *C. difficile* test and the amplification of the triose phosphate isomerase (*tpi*) gene by PCR. Table 2 illustrates the characteristics of toxin genes and antimicrobial resistance profiles of the environmental *C. difficile* isolates.

Fifty percent of the farm samples were positive for *C. difficile* in cattle feces and a correlation between the age of the cattle/calves and the detection of *C. difficile* in feces could be observed. The occurrence of *C. difficile* in calf feces and the antimicrobial prescriptions on farms are shown in Table 1. *C. difficile* was found in the feces of calves that were treated with paromomycin, amoxicillin–colistin (farm 6), or spectinomycin–lincomycin (6/6, 100%), (3/6, 50%), or (1/3, 33.33%), respectively, while it was not observed in the analyzed calf feces treated with sulphanilamide–neomycin.

### 2.2. Toxin-Encoding Genes of Environmental C. difficile Strains

Environmental *C. difficile* isolates were screened for toxin genes (*tcdA* and *tcdB*) and binary toxins (*cdtA* and *cdtB*) via a multiplex PCR assay. Almost all isolates were toxigenic, with 88.76% positive samples for both toxin A (*tcdA*) and B (*tcdB*). There were 75 isolates (44.38%) positive for both binary toxins CDT (*cdtA* and *cdtB*). All those isolates were positive for both toxins A and B. Nineteen isolates (11.24%) were non-toxigenic (Table 2). The highest number of *C. difficile* toxigenic isolates was recovered from a WWTP (55, 32.54%) and from calf feces (40, 23.67%).

### 2.3. Antimicrobial Resistance of Environmental C. difficile Strains

The susceptibility of environmental *C. difficile* isolates to six antimicrobials was determined by the disc diffusion method and the minimum inhibitory concentrations (MICs) by using an E-test. All environmental *C. difficile* isolates (*n* = 169) were susceptible to the antimicrobials vancomycin and metronidazole (Table 2). Most isolates (66.86%, *n* = 113) were resistant to ciprofloxacin, followed by clindamycin, moxifloxacin, and tetracycline, with 46.15% (*n* = 78), 13.02% (*n* = 22), and 4.73% (*n* = 8), respectively. Seventy-five (44.38%) out of one-hundred-sixty-nine isolates displayed resistance to at least two of the antimicrobials. Ninety-four (62.67%) and sixty-two (41.33%) out of one-hundred-fifty toxigenic isolates were resistant to ciprofloxacin and clindamycin, respectively. Furthermore, all non-toxigenic isolates were resistant to ciprofloxacin, while 15 isolates were resistant to clindamycin, 1 isolate was resistant to moxifloxacin, and 1 was resistant to tetracycline.

### 2.4. Standard Curves, Limit of Detection, and Detection Accuracy of qPCR for the 16S rRNA Gene

**Figure 1:**
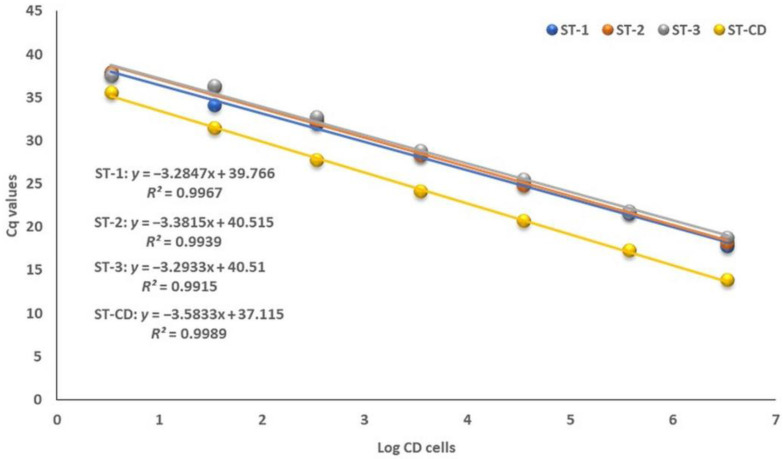
Amplification efficiencies of standards of pure culture of *C. difficile* DSM 1296 (ST-CD) and triplicate assays of CD-spiked feces (ST-1, ST-2, and ST-3).

Serial dilutions of *C. difficile* DSM strain 1296 (CD) from 10^−1^ to 10^−7^ (from 3.4 × 10^6^ to 0.34 CD cells) were spiked in CD-negative feces, and three standard curves were performed as described in Materials and Methods. Moreover, the standard curve was performed with *C. difficile* DSM 1296 pure culture. The quantification cycles (Cq) in 7 log dilutions ranged from 17.80 to 37.94 for three standards with *R*^2^ of 0.9967, 0.9939, 0.9915, while Cq for the analytical standard of the pure culture of *C. difficile* ranged from 13.87 to 35.64 with *R*^2^ of 0.9989 (Figure 1). The two analytical standard curves were performed to evaluate the quantitative detection accuracy and the limit of detection between the pure culture of *C. difficile* DSM strain 1296 and CD-spiked feces. The analytical curves of the pure culture of *C. difficile* DSM strain 1296 and CD-spiked feces for the 16S rRNA gene had almost equal slopes. These results confirmed that the TaqMan-based qPCR method was capable of detecting the target “*C. difficile*” in pure culture and in CD-spiked feces with high accuracy. These results also indicated that a TaqMan-based qPCR assay was qualified to quantify the *C. difficile* in feces with a low detection limit of 22.66 cells/g of feces.

The intra-assay CVs of the three standards were between 0.11% and 5%, 0.11% and 5.28%, and 0.11% and 5.69%, whereas the inter-assay CVs of the three standards ranged between 2.25% and 5.76, 3.05% and 5.36%, and 3.19% and 5.31%.

### 2.5. Quantification of Environmental C. difficile in Fecal Environmental Samples

A load of *C. difficile* cells was estimated by TaqMan-based PCR assay for 16S rRNA gene with DNA extracted from the 81 fecally contaminated environmental samples, as described in Table 1. In total, 45 out of 81 samples (55.56%) were positive for the *C. difficile* 16S rRNA gene, with counts ranging from 0.044 to 1561.62 cells per g or mL (Table 3, Table S1 in Supplementary Materials), and 36 samples were negative or under the detection limit (44.44%). *C. difficile* was mainly detected in 14 samples derived from the feces of calves and WWTP samples, and it was also detected in the samples of soil, digested sludge-amended soils, digested raw sewage sludge, and horse feces.

### 2.6. Comparison of Environmental C. difficile Detection by qPCR and C. difficile Selective Enrichment Culture in Fecally Contaminated Environmental Samples

**Figure 2:**
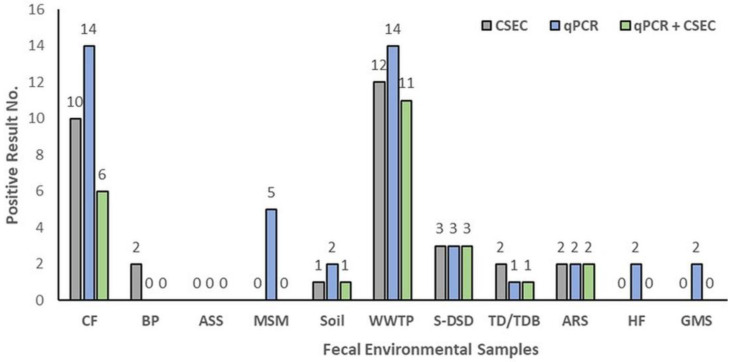
Comparison of detection results of environmental *C. difficile* between qPCR and *C. difficile* selective enrichment culture (CSEC). CF: calf feces; BP: biogas plant; ASS: activated sewage sludge; MSM: mixed storage manure; S-DSD: digested sludge-amended soils; TD/TDB: thermophilic digesters for treating sewage sludge or biowaste; ARS: anaerobic lab-scale bioreactors of sewage sludge digestion; HF: horse feces; GMS: grass and maize silage.

Results of *C. difficile* detection in fecally contaminated environmental samples by TaqMan-based qPCR assay were compared with those derived by *C. difficile* selective enrichment culture (CSEC). A total of 81 environmental samples were examined with both methods. Environmental *C. difficile* was detected in 45 of the 81 samples (55.56%) by qPCR, whereas *C. difficile* was isolated by CSEC from 32 samples (39.50%) (Figure 2, Table S2 in Supplementary Materials). *C. difficile* was positive for both qPCR and CSEC in 24 samples (75%), while it was negative in 28 samples (57.14%) (Table 4).

Eight confirmed enrichment culture-positive samples appeared to be negative by qPCR (Table 4), which could be explained by the lower target concentrations, meaning that the detection limit consists of less than 10 copies of the target DNA per PCR reaction [31,32,33]. Moreover, this might be related to the DNA extraction method and the increase in PCR inhibitors in those fecal samples. In addition, the DNA extraction efficiency from spores was approximately 1000 times lower than the efficiency when DNA was extracted from vegetative cells [28]. Additionally, among the 49 CSEC-negative samples that were not in concordance, 21 (42.86%) samples were positive with qPCR but not with an enrichment culture.

In those fecal samples, environmental *C. difficile* was not found with selective enrichment culture. This could be referred to as the used selective medium containing antimicrobial agents, the size of the sample, and other supplements. However, from the 49 CSEC-negative samples, only 28 (57.14%) samples gave the same result in the qPCR assay (Table 4).

## 3. Discussion

*C. difficile* is responsible for antibiotic-associated diarrhea in humans, and it was suggested that environmental sources outside healthcare institutions, such as animal feces, manure, wastewater, and sewage sludge from WWTPs [9,20,21,22,23,34], play a crucial role as a reservoir of community-associated *C. difficile* infections. The prevalence of *C. difficile* was found in different environments, such as animal farms [9,20,35,36,37,38], anaerobically digested sewage sludge from WWTP [20,39], animal manure and compost [21], soil [20], and vegetables, lawn and compost [40,41]. To the best of our knowledge, this is the first study that represents the prevalence, antimicrobial susceptibility patterns, toxin-encoding genes, and quantitative numbers of environmental *C. difficile* in various environmental samples contaminated by feces in a limited geographical region in Germany.

By using selective enrichment, positive results could be obtained from different sources such as WWTP samples (75%), soil (50%), and the feces of calves (31.25%), and the values are in agreement with the previously reported *C. difficile* occurrence from animal farms (4.3% to 36%) [9,36,42,43], WWTP samples (27% to 100%) [21,39,44], and soil (3% to 79) [18,19,42,43,45]. Additionally, this is the first report on the occurrences of *C. difficile* in digested sludge-amended soils, biogas plants, lab-scale bioreactors for the anaerobic thermophilic digestion of sewage sludge (control and experiment), and thermophilic digesters treating sewage sludge or biowaste with 100%, 100%, 100%, and 66.67% of positive samples, respectively. As a consequence, the disposal of the feces and manure of animals, biogas plant-derived, thermophilic digester-derived products, and digested sewage sludge as fertilizer on agricultural land could lead to environmental contamination with *C. difficile* spores, which may survive under adverse environmental conditions. Contaminated vegetables, meat products, or water might thus represent another indirect transmission pathway of CDI [20,41,46,47,48,49].

In this study, the frequency of the detection of toxigenic strains was high (88.76%), especially in isolates that recovered from WWTP samples and the feces of claves and, in consequence, must be considered completely virulent and able to cause antibiotic-associated diarrhea and pseudomembranous colitis in humans. The toxigenic strains of *C. difficile* were previously isolated from animal manure and compost [21], poultry manure, soil, dust [22], the feces of calves [9,36], and WWTP samples [39]. The present study and some previous studies confirmed that those sources also carry both toxigenic and antimicrobial-resistant *C. difficile* isolates. In our study, toxigenic *C. difficile* isolates were resistant to ciprofloxacin and clindamycin by 62.67% and 41.33%, respectively.

Interestingly, the presence of environmental *C. difficile* was observed in the feces of calves that were treated with antimicrobials such as paromomycin (belonging to the aminoglycoside class) or combined antimicrobials (amoxicillin (belonging to penicillins) and colistin (belonging to polymyxins) as well as spectinomycin (belonging to the aminocyclitol class) and lincomycin (belonging to lincosamide class)) on farms with positive results of *C. difficile* in 100%, 50%, and 33.33% of the samples, respectively. *C. difficile* was not observed in calf feces treated with combined antimicrobials (sulphadiazine (belonging to sulfonamides) and neomycin (belonging to the aminoglycoside class)), probably due to their low utilization on farms. It should be noted that the administration of antimicrobials to individual calves and the fecal shedding of *C. difficile* from the same calf could be directly linked. It was also observed that *C. difficile* could be detected in feces after penicillin prescriptions on the farm [9]. In humans, penicillins were reported as being associated with *C. difficile* infections [8]. Moreover, it could be identified that prior antimicrobial treatment increases the frequency of *C. difficile* fecal shedding from calves.

In the present study, the number of ciprofloxacin resistance (2nd generation of fluoroquinolones) in environmental *C. difficile* isolates obtained from various environmental samples was 66.86%. The number of moxifloxacin resistance (3rd generation of fluoroquinolones) in these isolates was 13.02%. Recently, a large number of *C. difficile* isolates were found that expressed a higher resistance to the 2nd generation of fluoroquinolones (ciprofloxacin) than to the 3rd generation of fluoroquinolones (moxifloxacin) [11,12,40]. Fluoroquinolone resistance in *C. difficile* strains occurs via mutations in the quinolone resistance-determining region (QRDR) of DNA gyrase subunits A (*gyrA*) and/or B (*gyrB*), resulting in several amino acid substitutions that confer resistance to fluoroquinolones [11,50].

Clindamycin belongs to the lincosamide class. Clindamycin resistance was discovered in 46.15% of all environmental *C. difficile* isolates in this study. Clindamycin resistance was reported in *C. difficile* isolates from different environmental sources, such as the feces of dairy calves (76.5%) [9], manure and compost samples (53.45%) [21], vegetables, lawn, and compost (33.6%) [40], swine and dairy feces (79.5 %) [37], and puddle water and soils (28.6%) [19]. Nineteen and fifteen non-toxigenic strains, classified as non-virulent, were resistant to ciprofloxacin and clindamycin, respectively. One isolate was resistant to tetracycline, and another one was resistant to moxifloxacin. These multiple antimicrobial resistances in non-toxigenic environmental *C. difficile* strains might serve as reservoirs of antimicrobial resistance determinants, which may be horizontally transferred to toxigenic strains, as well as into other pathogenic bacterial species via horizontal gene transfer (HGT).

The environmental *C. difficile* isolates recovered from raw sewage, calf feces, anaerobically digested sludge, and digested sludge-amended soils were resistant to tetracycline by 4.73%, which is comparable to the already published studies of clinical and environmental *C. difficile* isolates such as *C. difficile* isolates from soil and water (8.6%) [19] and vegetables, lawn, and compost (2.9%) [40]. In *C. difficile*, resistance to tetracycline is encoded by tetracycline (*tet*) resistance genes. The most widespread *tet* gene is *tetM*, usually associated with conjugative transposons Tn*916*/Tn*916*-like family and Tn*5397*. These elements are found to be able to transfer the *tet* genes among *C. difficile* strains and between unrelated species of bacteria present in the clinical setting, community, and in the environment, including animal reservoirs, food sources, soil, and water [7].

*C. difficile* resistance to antimicrobial agents (i.e., fluoroquinolones, macrolide–lincosamides–streptogramin B (MLS_B_), tetracyclines, or beta-lactams) could be a result of the presence of antimicrobial resistance genes (ARGs) via the transfer of mobile genetic elements (e.g., plasmids, conjugative transposons, prophages), occurrences of gene mutations, and changes in the antimicrobial targets and/or metabolic pathway of *C. difficile* and via biofilm formation [7,11,12,50,51]. HGT plays a key role in the spread of ARGs among toxigenic and non-toxigenic *C. difficile* strains and between other gut microbiota [52,53].

Culture-independent approaches with targets on the bacterial 16S rRNA gene have come into prominence for the detection and quantification of anaerobic fecal bacterial species, practically those present in relatively small numbers, such as *C. difficile* and *C. perfringens*, compared to the dominant gastrointestinal bacterial flora in animals and humans. In such cases, a selective enrichment culture is necessary, but it is time- and lab-consuming. Therefore, a TaqMan real-time qPCR assay for the rapid detection of the 16S rRNA gene of environmental *C. difficile* was used directly with the DNA extracted from the diverse fecally contaminated environmental samples, and a comparison with the results of the *C. difficile* selective enrichment culture method was performed.

To our knowledge, this is the first study that quantitatively evaluated numbers of environmental *C. difficile* in different environmental sources contaminated by feces and compared these with results from *C. difficile* selective enrichment cultures. Several studies, however, used qPCR to qualitatively and quantitatively determine the occurrence of *C. difficile* in clinical samples [24,28,29,54]. The fecally contaminated environmental sources outside healthcare institutions (i.e., WWTP samples, cattle feces, soil, digestion of raw sewage sludge, horse feces) could directly or indirectly spread *C. difficile* in the community [9,21,35,36,37,39] and may be a potential health risk.

In this study, a TaqMan-based qPCR assay was qualified to quantify *C. difficile* in feces with a low detection limit of 22.66 cells per g of feces, which is slightly higher than in other published studies to date. Bandelj et al. [25] and Balamurugn et al. [55] published a detection limit of approximately 7.72 and 10 *C. difficile* cells per g of feces, respectively. Contrarily, Rintilla et al. [56], Penders et al. [24], and Kubota et al. [28] obtained higher detection limits between 6 × 10^3^ and 6 × 10^4^ of *C. difficile* cells, 2 × 10^3^ CFU/g of feces, and 10^3^ cells per g stool, respectively. In addition, the detection limit of *C. difficile* toxin genes (*tcdA* and *tcdB*) was found to be 5 × 10^4^ CFU/g of feces [57]. The highest numbers of *C. difficile* were found in digested sludge-amended soils, digested sewage sludge, the feces of calves, anaerobic lab-scale bioreactors for the thermophilic digestion of sewage sludge, and soils, ranging between 4.4 × 10^1^–2.67 × 10^2^, 1.2 × 10^2^–7.61 × 10^2^, 1.6 × 10^1^–8.2 × 10^2^, 8.15–1.75 × 10^3^, 4.7 × 10^1^–2.08 × 10^2^, and 1.49 × 10^1^–3.75 × 10^2^ cells per g or mL of fecal sample, respectively. The numbers of *C. difficile* in the feces of calves are in agreement with the numbers from a recent study that reported that *C. difficile* was found in cattle feces with counts ranging from 2.87 × 10^2^ to 2.65 × 10^4^ cells per g [25].

In our examinations, the reliable detection of *C. difficile* in different fecally associated samples and the comparison of results obtained between CSEC and qPCR methods (Table S1) supports the validity of TaqMan qPCR as a sensitive method to detect *C. difficile* in fecal environmental samples. *C. difficile* was detected in 45 out of 81 samples (55.56%) via qPCR, whereas it was detected in 32 samples (39.50%) by selective enrichment culture. Brown et al. [27] reported that the *C. difficile* 16S rRNA gene was detected in 64.6% and 43.8% of environmental surface area by qPCR and enrichment culture, respectively. However, the results obtained with qPCR correlate with the selective enrichment cultures in 24 (75%) samples, but qPCR was more sensitive and able to detect *C. difficile* in 21 enrichment culture-negative cases. Eight enrichment culture-positive samples were qPCR negative (Table 4). This might relate to the number of *C. difficile* cells or spores in fecal samples. In addition, the used DNA extraction method may reduce the target concentration, meaning that the sample consists of less than 10 copies of the target DNA [31,32,33]. In our study, the DNA concentration ranged between 1.08 and 384 ng/µL, and the DNA template was subjected to qPCR with or without dilution. In general, the DNA extraction from fecal samples and the resuspension in smaller amounts of elution buffer could not only give highly concentrated DNA but also increase fecal-derived PCR inhibitors and decrease the efficiency of amplification [28,58]. Kubota et al. [28] also reported that the qPCR assay mainly detected vegetative cells because the DNA extraction efficiency from spores was approximately 1000 times lower than the efficiency from vegetative cells. The expected reasons for not detecting *C. difficile* via 16S rRNA gene qPCR or in enrichment cultures are summarized in Table 5.

Among the 49 CSEC-negative samples, 21 samples were qPCR-positive. The discrepant result between the selective enrichment culture and TaqMan qPCR methods in the 21 samples may reflect that the selective enrichment culture method detects only living cells; qPCR detects both living and dead cells, which could result in a higher detection frequency of *C. difficile* by a TaqMan qPCR assay (Table 5). The quantification of the 16S rRNA gene by real-time qPCR in antibiotic-associated diarrhea patients was correlated with the culture, but qPCR was more sensitive and able to detect *C. difficile* in some culture-negative samples [54]. The low detection limit in *C. difficile*-spiked human stool samples by traditional PCR was 10-fold higher than the LOD from the culture method [33]. Moreover, used medium type, sample size, and selective supplemented agents (e.g., antimicrobials) might contribute to the apparent variation in *C. difficile* prevalence in those samples by using the enrichment culture method. The higher sensitivity found by qPCR was expected due to the detection of non-cultivable cells or spores. Additionally, the 16S rRNA gene qPCR and selective enrichment culture methods are all acceptable techniques for the detection and quantification of environmental *C. difficile*, but the qPCR assay is more sensitive than the selective enrichment culture.

## 4. Materials and Methods

### 4.1. Fecal Environmental Samples Collection

Eighty-one fecally contaminated environmental samples were collected from March 2021 to June 2022, including cattle feces, soil, digested sludge-amended soils, mixed storage cattle manure, horse feces, thermophilic digesters of biowaste or sewage sludge, biogas plant, anaerobic lab-scale bioreactors for thermophilic digestion of sewage sludge, and samples from a WWTP, located in northwestern Germany, including raw sewage (influent), treated sewage (effluent), activated sewage sludge, raw sewage sludge (mixture of activated sewage sludge and access of secondary sedimentation), and digested sewage sludge. The fecal environmental samples are summarized in Table 1.

### 4.2. Isolation and Identification of C. difficile from Fecally Contaminated Environmental Samples

One to three grams or mL of cattle feces, digested sludge-amended soils, raw sewage sludge, digested sewage sludge, and content of thermophilic digesters or biogas plant were inoculated in 9 mL *Clostridium difficile* selective (CD) broth, which consists of proteose peptone 40 g/L, fructose 6.0 g/L, Na_2_HPO_4_ 5.0 g/L, KH_2_PO_4_ 1.0 g/L, MgSO_4_·7H_2_O 0.1 g/L, NaCl 2.0 g/L. The broth was supplemented with (12 mg/L) norfloxacin (Sigma-Aldrich Chemie GmbH, Taufkirchen, Germany) and (32 mg/L) moxalactam (Biomol GmbH, Hamburg, Germany) and 0.1% sodium taurocholate (Carl Roth GmbH & Co. KG, Karlsruhe, Germany) for spore germination. All inoculated CD broths were prepared anaerobically in an anaerobic chamber (Coy Laboratory Products, Inc. Los Angeles, CA, USA) and flushed with a mixture of gases (80% N_2_ and 20% CO_2_). All inoculated CD broths were incubated at 37 °C for 7–10 days.

For raw sewage (influent), activated sewage sludge, and treated sewage (effluent), 100 or 300 mL of the sewage-derived samples were centrifuged at 10,000× *g* for 10 min at 4 °C, the supernatant was discarded, and the pellet was resuspended in one milliliter of CD broth. Afterward, the mixture was inoculated into supplemented CD broths and incubated as described above. For soil samples, soil was processed as described previously by Janezic et al. [19] with some modifications. Briefly, 25 g of soil was resuspended in 90 mL of sterile distilled water. In order to remove the majority of soil particles, 50 mL of soil suspension was centrifuged at 50× *g* for 2 min. Of soil suspension, 40 mL was transferred into a new 50 mL sterile centrifugation tube and centrifuged again at 50× *g* for 2 min. Of the supernatant, 30 mL was centrifuged at 10,000× *g* for 10 min, the supernatant was discarded, and the pellet was inoculated in 9 mL of supplemented CD broth. All inoculated broths were incubated as described above.

For grass and maize silage and horse feces, five grams of each sample were vortexed in 15 mL of 1× phosphate-buffered saline (PBS) for 1 min, three times. The collected suspensions were centrifuged at 10,000× *g* for 10 min at 4 °C, the supernatant was discarded, and the pellets were inoculated in 9 mL supplemented CD broths and incubated as mentioned above.

Following incubation, 2 mL of each incubated CD broth was mixed with an equal amount of absolute alcohol (1:1) and incubated at room temperature for 50–60 min. The mixtures were then centrifuged at 4000 rpm for 10 min, and the supernatant was discarded. The pellet was resuspended in 200 μL 1× PBS. All resuspended liquid or at least 100 µL was plated on *Clostridium difficile* agar basis (CDA, Fisher Scientific GmbH, Schwerte, Germany) supplemented with 7% defibrinated horse blood (Fisher Scientific GmbH, Schwerte, Germany), (12 mg/L) norfloxacin, (32 mg/L) moxalactam, and 0.1% sodium taurocholate. All plates were incubated anaerobically in anaerobic jars (Schuett-Biotec GmbH, Göttingen, Germany) at 37 °C for two days and, if negative, re-incubated three days more. Of each plate suspected of being *C. difficile*, 5–10 colonies (based on morphology, grey with irregular edges) were carefully picked and streaked onto CDA or blood agar supplemented with 5% horse blood and incubated anaerobically at 37 °C for 48 h. The identity of the pure culture was evaluated on the basis of morphology and confirmed via the Oxoid *C. difficile* latex test (Fisher Scientific GmbH, Schwerte, Germany) and finally by analyzing the *tpi* gene (see below in Section 4.4). Stock cultures of confirmed *C. difficile* isolates were stored in brain heart infusion (BHI) broth (Carl Roth GmbH & Co. KG, Karlsruhe, Germany) with 20% glycerol at −20 °C.

### 4.3. Genomic DNA Extraction from Bacterial Cells (Pure Cultures)

*C. difficile* colonies were transferred to 150–200 µL of 5% Chelex 100 in TE buffer (10 mM Tris-HCl, 1 mM EDTA, pH 8), pre-heated at 56 °C for 30 min. Afterwards, samples were boiled at 95 °C for 15 min with gentle vortexing every 5 min under continuous shaking at 300 rpm. The tube was centrifuged at high speed at 12,000× *g* for 3 min to pellet the Chelex. The supernatant (approximately 130–180 µL) containing the eluted genomic DNA was transferred to a new 1.5 mL Eppendorf tube. The eluted genomic DNA was centrifuged again for 3 min as described above, and 100–150 µL was removed and transferred to a final 1.5 mL Eppendorf tube. The genomic DNA was stored at −20 °C for further analysis. The genomic DNA was diluted 1:10 in MQ water, and five microliters (DNA concentration ranged between 1 and 1.5 ng/µL) were used directly in PCRs as DNA templates.

### 4.4. Molecular Identification of Environmental C. difficile Isolates via PCR

PCR amplification of a specific housekeeping gene, triose phosphate isomerase *(tpi)* was performed as previously described by Leeme et al. [59]. The PCR was performed with *tpi*-specific primers (tpi-F: AAAGAAGCTACTAAGGGTACAAA) and (tpi-R: CATAATATTGGGTCTATTCCTAC), with an amplicon size of 230 bp. The *C. difficile* DSM (Leibniz Institute, German Collection of Microorganisms, Braunschweig, Germany) strain 1296 was used as a positive control. PCR products were run under standard conditions on a 1% agarose gel and stained with a DNA stain (SERVA Electrophoresis GmbH, Heidelberg, Germany), and visualized under UV light.

### 4.5. Profiling of Toxin-Encoding Genes of Environmental C. difficile Isolates by Multiplex PCR

Amplification of toxin genes (*tcdA* and *tcdB*) and binary toxin genes (*cdtA* and *cdtB*) were detected using a multiplex PCR, as described previously by Perrson et al. [60]. The primers are listed in Table 6. *C. difficile* DSM 1296 was used as a positive control for toxin genes, *tcdA* and *tcdB*, but negative for binary toxin genes, *cdtA* and *cdtB*. In addition, one of our *C. difficile* strain was sequenced with an Illumina MiSeq in order to confirm the presence of the respective toxin genes which used as a positive control for those genes. PCR products were analyzed by electrophoresis on a 1.5% agarose gel.

### 4.6. Antimicrobial Susceptibility Testing

Environmental *C. difficile* isolates were subjected to antimicrobial susceptibility testing by the disc diffusion method for the antimicrobials clindamycin, ciprofloxacin, and tetracycline (Fisher Scientific GmbH, Schwerte, Germany). The minimum inhibitory concentrations (MICs) were determined by using an E-test of the antimicrobials vancomycin, metronidazole, and moxifloxacin (bioMe’rieux Deutschland GmbH, Nürtingen, Germany). The moxifloxacin’s concentration tested was 0.002–32 µg/mL. For vancomycin and metronidazole, the range tested was 0.016–256 µg/mL. The environmental *C. difficile* isolates were streaked on blood agar plates and were incubated anaerobically at 37 °C for 24 h. The inoculum was prepared by picking a few colonies and mixing them in two milliliters of physiological saline (0.85% NaCl). A bacterial suspension equivalent to 4 MacFarland units [61] was spread on Brucella agar plates using a sterile cotton swab (Sigma-Aldrich Chemie GmbH, Taufkirchen, Germany) supplemented with hemin and vitamin K, according to Clinical and Laboratory Standards Institute (CLSI) [62] for the testing of anaerobes. Antimicrobial discs and E-test strips were placed onto agar plates. The plates were incubated anaerobically for 24–48 h at 37 °C. For the disks, the diameter of the inhibition zone was measured. For the E-test, the MIC value was read from the scale in terms of µg/mL where the ellipse edge intersects the strip. The breakpoint/epidemiological cut-off of the E-test was interpreted according to the European Committee on Antimicrobial Susceptibility Testing (EUCAST) [63] guideline for vancomycin. The breakpoints of metronidazole and moxifloxacin were interpreted according to CLSI guidelines [62]. The inhibition zone diameter breakpoints of clindamycin were interpreted according to the members of the SFM Antibiogram Committee [64], while ciprofloxacin and tetracycline were interpreted according to Kouassi et al. [65].

### 4.7. Preparation and DNA Extraction from Fecal Environmental Samples

100–400 mg of fecal and soil samples were used for DNA extraction. Of raw and digested sewage sludge, thermophilic digesters content, storage mixed cow manure, and biogas plant digestate, 4 mL were centrifuged at 12,000× *g* for 5 min, the supernatant was discarded, and the pellet was used for DNA extraction as described above. For raw and treated sewage and activated sewage sludge, 35 to 300 mL of each was centrifuged at 10,000× *g* for 10 min at 4 °C, the supernatant was discarded, and the pellets were resuspended in provided buffer for DNA extraction. Grass and maize silage and horse feces were pre-treated, as described above in Section 4.1. 100–400 mg was weighted from the pellets, or the pellet was resuspended in provided buffer for DNA extraction. The DNA was extracted from fecal environmental samples by using Allprep^®^ PowerViral^®^DNA/RNA Kit (Qiagen, Hilden, Germany) or Quick-DNA™ Fecal/Soil Microbe Miniprep Kit (Zymo Research, Irvine, CA, USA) according to the respective protocols. The extracted DNA was stored at −20 °C until further analysis. The DNA concentration was quantified via Qubit 3.0 Fluorometer.

### 4.8. Preparation of Standard Analytic Curves of C. difficile-Spiked Feces and Pure Culture for qPCR

The standard analytic curves of *C. difficile* (CD) were performed as described previously by Bandelj et al. [25]. Briefly, the strain *C. difficile* DSM 1296 was cultured on brain heart infusion (BHI) agar plates. The plates were incubated anaerobically at 37 °C for 24 h. Afterward, the pure culture of CD was harvested from BHI agar plates into one milliliter of 1× PBS. 10-fold serial dilutions of CD stock suspension were prepared in 1× PBS (10^−1^ to 10^−7^). The number of *C. difficile* DSM 1296 cells was quantified by counting the cells with a microscope (Axioscope, Carl Zeiss Microscopy GmbH, Jena, Germany) using a Neubauer chamber (Marienfeld–Superior™ GmbH & Co.KG, Lauda-Königshofen, Germany). The number of CD cells per milliliter was calculated for dilutions, 10^−1^ to 10^−4^, according to the following equation:

Cells per mL = average count per square (from four squares) × dilution factor × 10^4^

Of the serial dilutions of the pure culture ranging from 3.4 × 10^7^ CD cells per mL to approximately 3.4 CD cells per milliliter, 100 µL was spiked in cattle feces. All serial dilutions were spiked in 150 mg cattle feces in triplicate that were previously confirmed by 16S rRNA gene-specific assay to be negative for *C. difficile* in genomic DNA directly extracted or after selective enrichment of cattle feces as well as by plating enrichment culture of cattle feces on *C. difficile* selective agar plates as described above in Section 4.1. The genomic DNA was isolated from the CD-negative feces spiked with a known number of CD cells and further tested in triplicate using the CD 16S rRNA gene TaqMan-based qPCR to generate a standard analytical curve. The genomic DNA was extracted from the CD-negative spiked feces by using Quick-DNA™ Fecal/Soil Microbe Miniprep Kit (Zymo Research, Irvine, CA, USA). The extracted genomic DNA was stored at −20 °C until further analysis.

Genomic DNA was extracted from 3.4 × 10^7^ cells of pure culture of *C. difficile* DSM 1296 with a Qiagen genomic DNA extraction kit (Qiagen, Hilden, Germany) using a silica-based kit (silica bead DNA extraction kit; Thermo Scientific, St. Leon-Rot, Germany). Its serial dilutions were applied to generate a standard analytical curve of the *C. difficile* DSM 1296 pure culture cells. The two standard analytical curves were compared and used to evaluate the lower detection limit and detection accuracy of this TaqMan-based qPCR assay. The DNA was used to generate CD genome qualification standards and to determine the amplification efficiency (Figure 1).

### 4.9. Quantitative Real-Time PCR Assay

Real-time quantitative PCR (qPCR) was carried out with primers (16S-F: TTGAGCGATTTACTTCGGTAAAGA) and (16S-R: TGTACTGGCTC ACCTTTGATATT CA), with amplicon size of 151 bp, and a TaqMan probe-16S rRNA (FAM-CCACGCGTTACTCACCCGTCCG) specific for *C. difficile* 16S rRNA gene [24]. Each reaction mixture of 25 µL for the qPCR assay composed of master mix, 10× standard reaction buffer, 0.2 mM dNTPs, 25 mM MgCl_2_, 0.2 µM of each specific primer, 0.2 µM of the TaqMan probe, 1.25 U of Hot Start *Taq* DNA polymerase (New England BioLabs GmbH), and 1 µL (undiluted) or 5 µL (diluted 1:10) of template DNA. The following PCR program was used for the amplification: 50 °C for 2 min, 95 °C for 10 min, followed by 45 cycles at 95 °C for 15 sec and 60 °C for 1 min. The assays were carried out with real-time PCR 5′-nuclease assays (TaqMan RT-PCR) in a MiniOpticon real-time PCR system (Bio-Rad, Hercules, CA, USA). Negative (water) and positive (*C. difficile* DSM 1296) controls were always included in each qPCR run.

### 4.10. Quantification of Environmental C. difficile in Fecally Contaminated Environmental Samples by TaqMan-Based qPCR Assay

Purified DNA from fecal samples is used to establish an appropriate standard curve to enumerate a load of *C. difficile* in the fecal samples. Fecal samples were analyzed in duplicate by qPCR from undiluted or diluted DNA, as mentioned above. The amount of DNA measured by qPCR was converted to cell numbers. This was accomplished by using the standard curve that was generated by plotting the Cq value against CD cell numbers corresponding to each DNA dilution (Figure 1). The intra- and inter-assay coefficient of variations (CVs) of the qPCR assay, PCR efficiency, and low detectable limits (LOD) were determined. The LOD was defined as the smallest CD cell number in each standard curve.

## 5. Conclusions

The environmental *C. difficile* strains are commonly present in various non-clinical sources, which could serve as a potential source of community-associated CDI. The specified TaqMan-based qPCR assay showed acceptable results with respect to detection limits, which makes this assay especially suitable for the rapid detection of *C. difficile* not only in patients and clinical environments but also in environmental sources outside healthcare institutions. The whole genome sequences of those environmental *C. difficile* strains are required to characterize virulence-associated factors or the genotypically antimicrobial resistance often located on mobile genetic elements (e.g., plasmids, conjugative transposons, prophages, insertion sequences). In addition, the epidemiological relatedness between clinical strains and those from non-clinical environments and animals needs further investigation.

## Figures and Tables

**Table 1:** Presence of *C. difficile* in diverse fecal environmental samples after selective enrichment.

Farm No./Sample ID	Number of Samples	Description/Medication	Sample Source	Age (Days)	Presence of *C. difficile*
Farm 1	3	Spectinomycin and Lincomycin	Calf feces	120	+(1/3)
Farm 2	3	Amoxicillin and Colistin	Calf feces	90	−(0/3)
Farm 3	3	Sulphanilamide and Neomycin	Calf feces	90	−(0/3)
Farm 4	3	Paromomycin	Calf feces	90	+(3/3)
Farm 5	2		Biogas plant	-	+(2/2)
Farm 6	3	Amoxicillin and Colistin	Calf feces	90	+(3/3)
Farm 7	3	Paromomycin	Calf feces	90	+(3/3)
Farm 8	4	Pooled cow feces	Cow feces	adult	−(0/4)
	3	Pooled calf feces	Calf feces	180	−(0/3)
	3	Pooled calf feces	Calf feces	90	−(0/3)
	2	Calves feeding with cow or artificial milk	Calf feces	90	−(0/2)
	2	Mixed storage manure	Mixed manure	-	−(0/2)
	1		Grass silage	-	−(0/1)
	1		Maize silage	-	−(0/1)
Farm 9	2	Cow manure (storage for 2 days)	Cow feces	adult	−(0/2)
	2	Cow manure (storage for 24 months)	Cow manure	adult	−(0/2)
	3	Pooled calf feces	Calf feces	120–240	−(0/3)
	1		Calf feces	42	−(0/1)
	1		Calf feces	90	−(0/1)
	1	Amoxicillin	Calf feces	30	−(0/1)
	2	Soil (collected from cattle farm)	Soil	-	+(1/2)
WWTP samples	4	Raw sewage (influent)			+(4/4)
	3	Treated sewage (effluent)			−(0/3)
	4	Raw sewage sludge			+(3/4)
	4	Digested sewage sludge			+(4/4)
	1	Activated sewage sludge			+(1/1)
S-DSD	1	S-DSD1: Soil treated for 10 years with digested sewage sludge, dried for one year			+(3/3)
	1	S-DSD2: Soil treated with digested sludge for 10 years but had not yet been dried			
	1	S-DSD3: Soil was in the process of being treated with digested sewage sludge			
TD-1	2	Thermophilic digester for treating sewage sludge			+(1/2)
TDB-1	1	Thermophilic digester for treating biowaste			+(1/1)
Control (C)	1	Anaerobic lab-scale bioreactor for thermophilic digestion of sewage sludge *^a^*			+(1/1)
Experiment (E)	1	Anaerobic lab-scale bioreactor for thermophilic digestion of sewage sludge + canola lecithin *^a^*			+(1/1)
HF	9	Horse feces			−(0/9)
Total	81				32 (39.50%)

WWTP: wastewater treatment plant; S-DSD: digested sludge-amended soils; TD: thermophilic digester for treating sewage sludge; HF: horse feces; TDB: thermophilic digester for treating biowaste. *^a^* The control and experimental samples were obtained from anaerobic lab-scale bioreactors for thermophilic digestion of sewage sludge with or without canola lecithin [30].

**Table 2:** Antimicrobial resistance and toxin gene profiles of environmental *C. difficile* isolates recovered from fecally contaminated environmental samples.

Isolate No.	Sample Source	Sampling Time	Toxin Genes	Binary Toxins	Antibiotic Resistance Profile ^1^
*C. difficile* (RSS1, RSS2, RSS3, RSS4, RSS5, RSS6, RSS7, and RSS10)	Raw sewage sludge	July 2021	*tcdA*, *tcdB*		
*C. difficile* (RSS11 and RSS12)					CIP, CLIN
*C. difficile* RSS13			*tcdA*, *tcdB*	*cdtAB*	
*C. difficile* (RSS37 and RSS52)	Raw sewage sludge	April 2021			CIP, CLIN
*C. difficile* RSS38			*tcdA*, *tcdB*		CIP
*C. difficile* RSS39			*tcdA*, *tcdB*		CIP, CLIN, MXF
*C. difficile* RSS61 and RSS68		December 2021	*tcdA*, *tcdB*	*ctdAB*	
*C. difficile* (RSS62, RSS63, RSS64, and RSS66)			*tcdA*, *tcdB*	*ctdAB*	CLIN
*C. difficile* RSS65			*tcdA*, *tcdB*	*ctdAB*	CIP
*C. difficile* RSS67			*tcdA*, *tcdB*	*ctdAB*	CIP, CLIN
*C. difficile* (RS8, RS14, and RS16)	Raw sewage	July 2021	*tcdA*, *tcdB*		
*C. difficile* RS9					CIP
*C. difficile* RS15			*tcdA*, *tcdB*	*cdtAB*	CIP, CLIN, TE
*C. difficile* RS17			*tcdA*, *tcdB*		CIP
*C. difficile* RS32		March 2021	*tcdA*, *tcdB*		CIP
*C. difficile* (RS35 and RS36)			*tcdA*, *tcdB*		CIP, CLIN
*C. difficile* (RS43 and RS44)		April 2021	*tcdA*, *tcdB*		CIP
*C. difficile* (RS147, RS149, RS150, RS151, RS152, and RS165)		May 2022			CIP, CLIN
*C. difficile* RS148			*tcdA*, *tcdB*		CIP
*C. difficile* (RS153 and RS154)			*tcdA*, *tcdB*		CIP, CLIN
*C. difficile* RS164					CIP, CLIN, TE
*C. difficile* DSS18	Digested sewage sludge	July 2021	*tcdA*, *tcdB*		CIP
*C. difficile* DSS19					CIP
*C. difficile* (DSS26, DSS27, DSS29, and DSS31)		March 2021	*tcdA*, *tcdB*		
*C. difficile* (DSS28 and DSS30)			*tcdA*, *tcdB*		CIP
*C. difficile* DSS41		April 2021	*tcdA*, *tcdB*		CIP
*C. difficile* (DSS183, DSS184, DSS185, DSS186, DSS187, DSS188, and DSS189)		June 2022	*tcdA*, *tcdB*		CIP, CLIN
*C. difficile* DSS190 and DSS191			*tcdA*, *tcdB*		CIP
*C. difficile* DSS202					CIP, CLIN
*C. difficile* ASS20	Activated sewage sludge	March 2021	*tcdA*, *tcdB*		CIP, CLIN
*C. difficile* (ASS21 and ASS22)			*tcdA*, *tcdB*		CIP
*C. difficile* (ASS23, ASS24, and ASS25)			*tcdA*, *tcdB*	*cdtAB*	CIP
*C. difficile* S45	Soil	August 2021	*tcdA*, *tcdB*		
*C. difficile* (CF69, CF70, CF76, CF77, CF83, CF107, CF129, CF193, CF195, and CF196)	Feces of calves	December 2021	*tcdA*, *tcdB*	*ctdAB*	CIP
*C. difficile* (CF72, CF74, CF78, CF81, CF88, CF89, CF90, CF91, CF101, CF102, CF113, and CF132)			*tcdA*, *tcdB*	*ctdAB*	
*C. difficile* (CF73, CF75, CF109, CF114, CF192, and CF194)			*tcdA*, *tcdB*	*ctdAB*	CIP, CLIN
*C. difficile* (CF79 and CF80)			*tcdA*, *tcdB*	*ctdAB*	CLIN
*C. difficile* (CF82, CF84, CF85, CF86, CF87, CF95, and CF97)	Feces of calves	December 2021	*tcdA*, *tcdB*	*ctdAB*	CIP, MXF
*C. difficile* CF99			*tcdA*, *tcdB*	*ctdAB*	CIP, CLIN, MXF
*C. difficile* CF92			*tcdA*, *tcdB*	*ctdAB*	TE
*C. difficile* CF103			*tcdA*, *tcdB*	*ctdAB*	CLIN, TE
*C. difficile* (BP71 and BP197)	Biogas plant	December 2021	*tcdA*, *tcdB*	*ctdAB*	CIP
*C. difficile* (BP198, BP199, and BP201)			*tcdA*, *tcdB*	*ctdAB*	CIP, CLIN
*C. difficile* (TDS115, TDS116, TDS120, and TDS121)	Thermophilic digester for treating sewage sludge	November 2021	*tcdA*, *tcdB*		
*C. difficile* (TDS119 and TDS122)			*tcdA*, *tcdB*		CIP, CLIN
*C. difficile* TDS117			*tcdA*, *tcdB*		CIP
*C. difficile* TDS118			*tcdA*, *tcdB*		CIP, CLIN, MXF
*C. difficile* TDS128			*tcdA*, *tcdB*	*ctdAB*	CIP
*C. difficile* (TDB123, TDB125, TDB126, TDB130, and TDB131)	Thermophilic digester for treating biowaste	November 2021	*tcdA*, *tcdB*		
*C. difficile* (TDB124 and TDB127)			*tcdA*, *tcdB*	*ctdAB*	
*C. difficile* (ARC134, ARC135, and ARC 182)	Anaerobic lab-scale bioreactors treating sewage sludge/control	April 2022	*tcdA*, *tcdB*		CIP
*C. difficile* (ARC139 and ARC166)			*tcdA*, *tcdB*		CIP, CLIN, MXF
*C. difficile* ARC140, ARC141, and ARC168)			*tcdA*, *tcdB*		CIP, CLIN
*C. difficile* ARC 167			*tcdA*, *tcdB*		TE
*C. difficile* ARE136	Anaerobic lab-scale bioreactors treating sewage sludge/experiment	April 2022	*tcdA*, *tcdB*		CIP
*C. difficile* (ARE137 and ARE170)			*tcdA*, *tcdB*		CIP, CLIN
*C. difficile* (ARE138, ARE143, and ARE144)			*tcdA*, *tcdB*		CLIN
*C. difficile* ARE 145					CIP
*C. difficile* ARE 146					CIP, CLIN
*C. difficile* (DS155, DS156, and DS175)	Digested sludge-amended soils	May 2022	*tcdA*, *tcdB*	*ctdAB*	CIP, CLIN
*C. difficile* DS157			*tcdA*, *tcdB*		CIP, CLIN, MXF
*C. difficile* (DS158, DS159, DS162, DS173, DS177, and DS181)			*tcdA*, *tcdB*	*ctdAB*	CIP, CLIN, MXF
*C. difficile* (DS160 and DS172)					CIP, CLIN
*C. difficile* DS161			*tcdA*, *tcdB*		CIP, CLIN, MXF, TE
*C. difficile* (DS163, DS169, and DS178)			*tcdA*, *tcdB*		CIP, CLIN
*C. difficile* DS171			*tcdA*, *tcdB*	*ctdAB*	MXF
*C. difficile* DS174			*tcdA*, *tcdB*	*ctdAB*	CIP, MXF
*C. difficile* DS176			*tcdA*, *tcdB*	*ctdAB*	CIP
*C. difficile* (DS179 and DS180)			*tcdA*, *tcdB*	*ctdAB*	CIP, CLIN, TE

^1^ CIP = Ciprofloxacin, CLIN = Clindamycin, MXF = Moxifloxacin, TE = Tetracycline.

**Table 3:** Quantification of environmental *C. difficile* from fecally contaminated environmental samples.

Sample Source	No. of CD Cells per g or mL	Sample Source	No. of CD Cells per g or mL
Digested sludge-amended soils	4.4 × 10^1^–2.67 × 10^2^	Thermophilic digester for treating biowaste	ND
Digested sewage sludge	1.2 × 10^2^–7.61 × 10^2 *a*^	Thermophilic digester for treating sewage sludge	4.7 × 10^1^–2.08 × 10^2 *a*^
Raw sewage	0.18–1.44	Soil (collected from cattle farm)	1.49 × 10^1^–3.75 × 10^2 *a*^
Treated sewage	0.044–0.49	Mixed storage cow manure	1.5 × 10^1^–1.96 × 10^1^
Raw sewage sludge	1.6 × 10^1^–2.03 × 10^1^ (8.2 × 10^2 *a*^)	Biogas plant	ND
Activated sewage sludge	3.4 × 10^1^	Adult cow feces	ND
Anaerobic lab-scale bioreactor digested sewage sludge	2.48 × 10^1^–3.06 × 10^1^	Grass and maize silage	0.49–1.12
Feces of claves	8.15–9.68 × 10^1^ (1.75 × 10^3 *a*^)	Horse feces	1.63 × 10^1^–6.39 × 10^1^

*^a^* values were calculated from 1:10 diluted samples. ND: not detected.

**Table 4:** Comparison of detected positive or negative results from qPCR assay with CSEC method.

Enrichment Culture Results	No. (%) of Samples with qPCR Results
Positive 32 (39.50%)	Positive 24 (75%)
	Negative 8 (25%)
Negative 49 (60.50%)	Positive 21 (42.86%)
	Negative 28 (57.14%)
Total of samples 81 (100%)	-

**Table 5:** Expected reasons for undetectable *C. difficile* via TaqMan qPCR assay or CSEC method in fecal environmental samples.

Detected by CSEC but Not via qPCR	Detected by qPCR but Not with CSEC
Lower concentration of the target gene. DNA extraction method. Fecal-derived PCR inhibitors (e.g., humic acids). DNA extraction efficiency from spores compares to vegetative cells. The sample with higher amount of DNA template also has the highest level of inhibitors that may inhibit the reaction.	Used media for isolation. Selective enrichment conditions. Selective agents (antimicrobials, e.g., moxalactam, norfloxacin, cefoxitin). Sample size. Other inhibitors in fecal sample, which might inhibit the growth of bacteria. The enrichment culture detects both living cells and spores, while qPCR detects living and dead cells.

**Table 6:** Primers for detection of toxin-encoding genes of *C. difficile*.

Target Gene	Amplicon Size (bp)	Primer Name	Sequence (5′-3′)
*tcdA*	629	tcdA-F3345	GCATGATAAGGCAACTTCAGTGGTA
		tcdA-R3969	AGTTCCTCCTGCTCCATCAAATG
*tcdB*	410	tcdB-F5670	CCAAARTGGAGTGTTACAAACAGGTG
		tcdB-R6079	GCATTTCTCCATTCTCAGCAAAGTA
*cdtA*	221	cdtA-F739	GGGAAGCACTATATTAAAGCAGAAGC
		cdtA-R958	CTGGGTTAGGATTATTTACTGGACCA
*cdtB*	262	cdtB-F617	TTGACCCAAAGTTGATGTCTGATTG
		cdtB-R878	CGGATCTCTTGCTTCAGTCTTTATAG

## Data Availability

Not applicable.

## Supplementary Materials

The following supporting information can be downloaded at: https://www.mdpi.com/article/10.3390/antibiotics12010162/s1, Table S1: Quantification of *C. difficile* from fecally contaminated environmental samples; Table S2: Comparison of detection results of Environmental *C. difficile* between qPCR and *C. difficile* selective enrichment culture (CSEC).

## References

[1] Crobach M.J.T., Vernon J.J., Loo V.G., Kong L.Y., Péchiné S., Wilcox M.H., Kuijper E.J. (2018). Understanding *Clostridium difficile* Colonization. Clin. Microbiol. Rev..

[2] Rupnik M., Wilcox M.H., Gerding D.N. (2009). *Clostridium difficile* infection: New developments in epidemiology and pathogenesis. Nat. Rev. Microbiol..

[3] Leffler D.A., Lamont J.T. (2015). *Clostridium difficile* Infection. N. Engl. J. Med..

[4] Dingle K.E., Elliott B., Robinson E., Griffiths D., Eyre D.W., Stoesser N., Vaughan A., Golubchik T., Fawley W.N., Wilcox M.H. (2014). Evolutionary history of the *Clostridium difficile* pathogenicity locus. Genome Biol. Evol..

[5] Gülke I., Pfeifer G., Liese J., Fritz M., Hofmann F., Aktories K., Barth H. (2001). Characterization of the enzymatic component of the ADP-ribosyltransferase toxin CDTa from *Clostridium difficile*. Infect. Immun..

[6] Davies K., Lawrence J., Berry C., Davis G., Yu H., Cai B., Gonzalez E., Prantner I., Kurcz A., Macovei I. (2020). Risk Factors for Primary *Clostridium difficile* Infection; Results From the Observational Study of Risk Factors for *Clostridium difficile* Infection in Hospitalized Patients With Infective Diarrhea (ORCHID). Front. Public Health.

[7] Peng Z., Jin D., Kim H.B., Stratton C.W., Wu B., Tang Y.-W., Sun X. (2017). Update on Antimicrobial Resistance in *Clostridium difficile*: Resistance Mechanisms and Antimicrobial Susceptibility Testing. J. Clin. Microbiol..

[8] Owens R.C., Donskey C.J., Gaynes R.P., Loo V.G., Muto C.A. (2008). Antimicrobial-Associated Risk Factors for *Clostridium difficile* Infection. Clin. Infect. Dis..

[9] Blasi F., Lovito C., Albini E., Bano L., Dalmonte G., Drigo I., Maresca C., Massacci F.R., Orsini S., Primavilla S. (2021). *Clostridioides difficile* in Calves in Central Italy: Prevalence, Molecular Typing, Antimicrobial Susceptibility and Association with Antibiotic Administration. Animals.

[10] Baines S.D., Wilcox M.H. (2015). Antimicrobial Resistance and Reduced Susceptibility in *Clostridium difficile*: Potential Consequences for Induction, Treatment, and Recurrence of *C. difficile* Infection. Antibiotics.

[11] O’Grady K., Knight D.R., Riley T.V. (2021). Antimicrobial resistance in *Clostridioides difficile*. Eur. J. Clin. Microbiol. Infect. Dis..

[12] Spigaglia P., Mastrantonio P., Barbanti F. (2018). Antibiotic Resistances of *Clostridium difficile*. Adv. Exp. Med. Biol..

[13] Camorlinga M., Sanchez-Rojas M., Torres J., Romo-Castillo M. (2019). Phenotypic Characterization of Non-toxigenic *Clostridioides difficile* Strains Isolated From Patients in Mexico. Front. Microbiol..

[14] Fraga E.G., Nicodemo A.C., Sampaio J.L.M. (2016). Antimicrobial susceptibility of Brazilian *Clostridium difficile* strains determined by agar dilution and disk diffusion. Braz. J. Infect. Dis..

[15] Aspevall O., Lundberg A., Burman L.G., Akerlund T., Svenungsson B. (2006). Antimicrobial susceptibility pattern of *Clostridium difficile* and its relation to PCR ribotypes in a Swedish university hospital. Antimicrob. Agents Chemother..

[16] Schmid A., Messelhäusser U., Hörmansdorfer S., Sauter-Louis C., Mansfeld R. (2013). Occurrence of zoonotic clostridia and Yersinia in healthy cattle. J. Food Prot..

[17] Hernandez B.G., Vinithakumari A.A., Sponseller B., Tangudu C., Mooyottu S. (2020). Prevalence, Colonization, Epidemiology, and Public Health Significance of *Clostridioides difficile* in Companion Animals. Front. Vet. Sci..

[18] Shivaperumal N., Chang B.J., Riley T.V. (2020). High Prevalence of *Clostridium difficile* in Home Gardens in Western Australia. Appl. Environ. Microbiol..

[19] Janezic S., Potocnik M., Zidaric V., Rupnik M. (2016). Highly Divergent *Clostridium difficile* Strains Isolated from the Environment. PLoS ONE.

[20] Rodriguez Diaz C., Seyboldt C., Rupnik M. (2018). Non-human *C. difficile* Reservoirs and Sources: Animals, Food, Environment. Adv. Exp. Med. Biol..

[21] Dharmasena M., Jiang X. (2018). Isolation of Toxigenic *Clostridium difficile* from Animal Manure and Composts Being Used as Biological Soil Amendments. Appl. Environ. Microbiol..

[22] Frentrup M., Thiel N., Junker V., Behrens W., Münch S., Siller P., Kabelitz T., Faust M., Indra A., Baumgartner S. (2021). Agricultural fertilization with poultry manure results in persistent environmental contamination with the pathogen *Clostridioides difficile*. Environ. Microbiol..

[23] Baghani A., Alimohammadi M., Aliramezani A., Talebi M., Mesdaghinia A., Douraghi M. (2020). Isolation and characterization of a multidrug-resistant *Clostridioides difficile* toxinotype V from municipal wastewater treatment plant. J. Environ. Health Sci. Eng..

[24] Penders J., Vink C., Driessen C., London N., Thijs C., Stobberingh E.E. (2005). Quantification of *Bifidobacterium* spp., Escherichia coli and *Clostridium difficile* in faecal samples of breast-fed and formula-fed infants by real-time PCR. FEMS Microbiol. Lett..

[25] Bandelj P., Logar K., Usenik A.M., Vengust M., Ocepek M. (2013). An improved qPCR protocol for rapid detection and quantification of *Clostridium difficile* in cattle feces. FEMS Microbiol. Lett..

[26] MacDougall L.K., Broukhanski G., Simor A., Johnstone J., Mubareka S., McGeer A., Daneman N., Garber G., Brown K.A. (2018). Comparison of qPCR versus culture for the detection and quantification of *Clostridium difficile* environmental contamination. PLoS ONE.

[27] Brown K.A., MacDougall L.K., Valenta K., Simor A., Johnstone J., Mubareka S., Broukhanski G., Garber G., McGeer A., Daneman N. (2018). Increased environmental sample area and recovery of *Clostridium difficile* spores from hospital surfaces by quantitative PCR and enrichment culture. Infect. Control Hosp. Epidemiol..

[28] Kubota H., Sakai T., Gawad A., Makino H., Akiyama T., Ishikawa E., Oishi K. (2014). Development of TaqMan-Based Quantitative PCR for Sensitive and Selective Detection of Toxigenic *Clostridium difficile* in Human Stools. PLoS ONE.

[29] Kohler C.M., Ana G., Randall T.H., Margolis E.B. (2022). Real-time quantitative PCR method for detection and quantification of *Clostridioides difficile* cells and spores. J. Microbiol. Methods.

[30] Jeske J.T., Gallert C. (2021). Mechanisms Driving Microbial Community Composition in Anaerobic Co-Digestion of Waste-Activated Sewage Sludge. Bioengineering.

[31] Ellison S.L.R., English C.A., Burns M.J., Keer J.T. (2006). Routes to improving the reliability of low level DNA analysis using real-time PCR. BMC Biotechnol..

[32] Gill P., Whitaker J., Flaxman C., Brown N., Buckleton J. (2000). An investigation of the rigor of interpretation rules for STRs derived from less than 100 pg of DNA. Forensic Sci. Int..

[33] Ganji L., Azimirad M., Farzi N., Alebouyeh M., Shirazi M.H., Eshraghi S.S., Mirshafiey A., Daryani N.E., Zali M.R. (2016). Comparison of the Detection Limits of the Culture and PCR Methods for the Detection of *Clostridium difficile*, Clostridium perfringens, Campylobacter jejuni, and Yersinia enterocolitica in Human Stool. Arch. Pediatr. Infect. Dis..

[34] Janezic S., Mlakar S., Rupnik M. (2018). Dissemination of *Clostridium difficile* spores between environment and households: Dog paws and shoes. Zoonoses Public Health.

[35] Janezic S., Ocepek M., Zidaric V., Rupnik M. (2012). *Clostridium difficile* genotypes other than ribotype 078 that are prevalent among human, animal and environmental isolates. BMC Microbiol..

[36] Redding L., Huang E., Ryave J., Webb T., Barnhart D., Baker L., Bender J., Kristula M., Kelly D. (2021). *Clostridioides difficile* on dairy farms and potential risk to dairy farm workers. Anaerobe.

[37] Thitaram S.N., Frank J.F., Siragusa G.R., Bailey J.S., Dargatz D.A., Lombard J.E., Haley C.A., Lyon S.A., Fedorka-Cray P.J. (2016). Antimicrobial susceptibility of *Clostridium difficile* isolated from food animals on farms. Int. J. Food Microbiol..

[38] Thitaram S.N., Frank J.F., Lyon S.A., Siragusa G.R., Bailey J.S., Lombard J.E., Haley C.A., Wagner B.A., Dargatz D.A., Fedorka-Cray P.J. (2011). *Clostridium difficile* from healthy food animals: Optimized isolation and prevalence. J. Food Prot..

[39] Xu C., Weese J.S., Flemming C., Odumeru J., Warriner K. (2014). Fate of *Clostridium difficile* during wastewater treatment and incidence in Southern Ontario watersheds. J. Appl. Microbiol..

[40] Lim S.-C., Androga G.O., Knight D.R., Moono P., Foster N.F., Riley T.V. (2018). Antimicrobial susceptibility of *Clostridium difficile* isolated from food and environmental sources in Western Australia. Int. J. Antimicrob. Agents.

[41] Bakri M.M., Brown D.J., Butcher J.P., Sutherland A.D. (2009). *Clostridium difficile* in ready-to-eat salads, Scotland. Emerg. Infect. Dis..

[42] Simango C. (2006). Prevalence of *Clostridium difficile* in the environment in a rural community in Zimbabwe. Trans. R. Soc. Trop. Med. Hyg..

[43] Båverud V., Gustafsson A., Franklin A., Aspán A., Gunnarsson A. (2003). *Clostridium difficile*: Prevalence in horses and environment, and antimicrobial susceptibility. Equine Vet. J..

[44] Romano V., Pasquale V., Krovacek K., Mauri F., Demarta A., Dumontet S. (2012). Toxigenic *Clostridium difficile* PCR Ribotypes from Wastewater Treatment Plants in Southern Switzerland. Appl. Environ. Microbiol..

[45] Gamboa M.d., Rodríguez E., Vargas P. (2005). Diversity of mesophilic clostridia in Costa Rican soils. Anaerobe.

[46] Candel-Pérez C., Ros-Berruezo G., Martínez-Graciá C. (2019). A review of Clostridioides *Clostridium difficile* occurrence through the food chain. Food Microbiol..

[47] Bauer M.P., Kuijper E.J. (2015). Potential sources of *Clostridium difficile* in human infection. Infect. Dis. Clin. N. Am..

[48] Gould L.H., Limbago B. (2010). *Clostridium difficile* in Food and Domestic Animals: A New Foodborne Pathogen?. Clin. Infect. Dis..

[49] Al Saif N., Brazier J.S. (1996). The distribution of *Clostridium difficile* in the environment of South Wales. J. Med. Microbiol..

[50] Wickramage I., Spigaglia P., Sun X. (2021). Mechanisms of antibiotic resistance of *Clostridioides difficile*. J. Antimicrob. Chemother..

[51] Mullany P., Allan E., Roberts A.P. (2015). Mobile genetic elements in *Clostridium difficile* and their role in genome function. Res. Microbiol..

[52] Smits W.K., Roseboom A.M., Corver J. (2022). Plasmids of *Clostridioides difficile*. Curr. Opin. Microbiol..

[53] Kartalidis P., Skoulakis A., Tsilipounidaki K., Florou Z., Petinaki E., Fthenakis G.C. (2021). *Clostridioides difficile* as a Dynamic Vehicle for the Dissemination of Antimicrobial-Resistance Determinants: Review and In Silico Analysis. Microorganisms.

[54] Naaber P., Stsepetova J., Smidt I., Rätsep M., Kõljalg S., Lõivukene K., Jaanimäe L., Löhr I.H., Natås O.B., Truusalu K. (2011). Quantification of *Clostridium difficile* in antibiotic-associated-diarrhea patients. J. Clin. Microbiol..

[55] Balamurugan R., Balaji V., Ramakrishna B.S. (2008). Estimation of faecal carriage of *Clostridium difficile* in patients with ulcerative colitis using real time polymerase chain reaction. Indian J. Med. Res..

[56] Rinttilä T., Kassinen A., Malinen E., Krogius L., Palva A. (2004). Development of an extensive set of 16S rDNA-targeted primers for quantification of pathogenic and indigenous bacteria in faecal samples by real-time PCR. J. Appl. Microbiol..

[57] Bélanger S.D., Boissinot M., Clairoux N., Picard F.J., Bergeron M.G. (2003). Rapid detection of *Clostridium difficile* in feces by real-time PCR. J. Clin. Microbiol..

[58] Pestana E.A. (2010). Early, Rapid and Sensitive Veterinary Molecular Diagnostics–Real Time PCR Applications.

[59] Lemee L., Dhalluin A., Testelin S., Mattrat M.-A., Maillard K., Lemeland J.-F., Pons J.-L. (2004). Multiplex PCR targeting *tpi* (triose phosphate isomerase), *tcdA* (Toxin A), and *tcdB* (Toxin B) genes for toxigenic culture of Clostridium difficile. J. Clin. Microbiol..

[60] Persson S., Torpdahl M., Olsen K.E.P. (2008). New multiplex PCR method for the detection of *Clostridium difficile* toxin A (*tcdA*) and toxin B (*tcdB*) and the binary toxin (*cdtA*/*cdtB*) genes applied to a Danish strain collection. Clin. Microbiol. Infect..

[61] Berger F.K., Mellmann A., von Müller L., Bischoff M., Gärtner B.C. (2020). Quality assurance for genotyping and resistance testing of *Clostridium* (Clostridioides) *difficile* isolates–Experiences from the first inter-laboratory ring trial in four German speaking countries. Anaerobe.

[62] Clinical and Laboratory Standards Institute (2021). Performance Standards for Antimicrobial Susceptibility Testing.

[63] The European Committee on Antimicrobial Susceptibility (2022). Clinical Breakpoints-Bacteria (Version 12.0).

[64] Members of the SFM Antibiogram Committee (2020). Comite’ de l’Antibiogramme de la Socie´te´ Francaise de Microbiologie (V.1.1 Avril).

[65] Kouassi K.A., Dadie A.T., N’Guessan K.F., Dje K.M., Loukou Y.G. (2014). Clostridium perfringens and *Clostridium difficile* in cooked beef sold in Côte d’Ivoire and their antimicrobial susceptibility. Anaerobe.

