# Supplementary material for: Prevalence, Antimicrobial Resistance and Toxin-Encoding Genes of Clostridioides difficile from Environmental Sources Contaminated by Feces
Source: Antibiotics (Basel). 2023 Jan 12;12(1):162. doi: 10.3390/antibiotics12010162 (PMC9855088; doi:10.3390/antibiotics12010162)
Supplement: Supplementary file 1 [file antibiotics-12-00162-s001.zip › antibiotics-2131796-supplementary.pdf]

antibiotics

Article

Prevalence, Antimicrobial Resistance and Toxin-Encoding Genes of *Clostridioides difficile* from Environmental Sources Contaminated by Feces

Khald Blau and Claudia Gallert *

Department of Microbiology–Biotechnology, Faculty of Technology, University of Applied Sciences Emden/Leer, 26723 Emden, Germany; khald.blau@hs-emden-leer.de
* Correspondence: claudia.gallert@hs-emden-leer.de

Table S1: Quantification of environmental *C. difficile* from faecally contaminated environmental samples.

Sample No.	Sample Source	Cq of 16S rRNA				Weight/Volume (g or mL)	Mean (Cq) Not diluted	Mean (Cq) Diluted 1:10	No. of CD cells per g or mL	
		Not diluted		Diluted (1:10)					Not diluted	Diluted × 10
S-DSD-1		ND	36.02	ND	40.79	0.20 g	36.02	40.79[a]	**44.60**	UDL
S-DSD-2	Digested sludge- amended soils	33.04	33.75	34.44	35.70	0.20 g	33.40	35.07	**267.73**	853.13
S-DSD-3		34.17	35.44	ND	38.30	0.20 g	34.81	38.30[a]	**102.24**	94.02
DSD-6-22		32.33	33.32	40.34	38.16	0.20 g	32.83	39.25[a]	**395.12**	103.45
DSD-7-21	Digested sewage sludge	ND	39.01	ND	ND	0.15 g	39.01[a]	-	UDL	-
DSD-4-21		38.32	39.14	33.07	35.81	0.15 g	38.73[a]	34.44	UDL	**761.40**
DSD-3-21		35.43	34.31	36.21	35.18	0.100 g	34.87	35.70	**120.47**	694.36
RS-5-22		31.28	30.96	32.1	34.82	100 mL	31.12	33.46	**1.44**	3.00
RS-3-21	Raw sewage	32.30	32.70	33.31	33.18	35 mL	32.50	33.22	**1.24**	7.44
RS-7-21		34.12	35.02	36.22	36.30	43 mL	34.57	36.26	**1.39**	4.50
RS-4-21		33.92	35.70	35.02	35.07	100 mL	34.81	35.05	**0.18**	1.56
TS-6-22		34.38	35.02	38.46	ND	300 mL	34.70	38.46[a]	**0.044**	UDL
TS-7-21	Treated sewage	ND	ND	ND	39.16	50 mL	-	39.16[a]	-	UDL
TS-4-21		33.15	32.36	36.72	35.15	100 mL	32.76	35.94	**0.49**	0.59
RSD-3-21		ND	ND	35.97	34.92	0.100 g	-	35.45	-	**820.53**
RSD-4-21	Raw sewage sludge	37.64	39.03	43.53[b]	37.46	0.15 g	38.34[a]	37.46	UDL	**94.77**
RSD-7-21		35.84	37.41	36.12	ND	0.15 g	36.63	36.12	**16.86**	238.89
RSD-12-21		36.62	38.95	34.74	33.51	0.20 g	36.62	34.13	**20.30**	1107.60
AS-3-21	Activated sewage sludge	35.59	n.d.	37.72	36.53	0.15 g	35.59	37.13	**34.46**	119.42
Control	Anaerobic lab-scale bioreactors	36.06	36.06	35.65	36.04	0.20 g	36.06	35.85	**30.68**	367.67
Experiment	for sewage sludge digestion	36.58	36.16	36.62	36.57	0.20 g	36.37	36.60	**24.85**	213.23
CF1	Faeces of claves	ND	ND	ND	ND	0.15 g	-	-	-	-
CF2		ND	ND	ND	ND	0.30 g	-	-	-	-

C3	ND	ND	ND	ND	0.15 g	-	-	-	-
CF4	ND	ND	ND	ND	0.15 g	-	-	-	-
CF5	ND	ND	ND	42.83	0.15 g	-	42.83[a]	-	UDL
C6	ND	ND	ND	ND	0.15 g	-	-	-	-
CF7	ND	ND	ND	ND	0.15 g	-	-	-	-
CF8	37.72	38.40	44.59	ND	0.15 g	38.06[a]	44.59[a]	UDL	UDL
CF9	ND	38.39	ND	ND	0.15 g	38.39[a]	-	UDL	-
CF10	37.15	36.23	38.93	37.96	0.15 g	36.69	38.45[a]	**96.87**	UDL
CF11	ND	ND	37.25	ND	0.15 g	-	37.25	-	**1561.62**
CF12	ND	ND	34.18	34.19	0.20 g	-	34.19	-	**1063.32**
CF15	ND	ND	37.36	ND	0.15 g	-	37.36	-	**1212.20**
CF16	ND	ND	ND	ND	0.30 g	-	-	-	-
CF17	ND	ND	37.20	ND	0.15 g	-	37.20	-	**1752.17**
CF18	38.92	39.55	36.44	36.52	0.40 g	39.24[a]	36.48	UDL	**111.66**

Table S1: Continued.

Sample No.	Sample Source	Cq of 16S rRNA				Weight/Volume (g or mL)	Mean (Cq) Not diluted	Mean (Cq) Diluted 1:10	No. of CD cells per g or mL	
		Not diluted		Diluted (1:10)					Not diluted	Diluted × 10
CF19	Faeces of claves	ND	ND	ND	ND	0.15 g	-	-	-	-
CF20		ND	ND	ND	38.89	0.15 g	-	38.89[a]	-	UDL
CF-1		ND	ND	ND	37.54	0.15 g	-	37.54	-	**186.83**
CF-2		ND	ND	ND	ND	0.15 g	-	-	-	**-**
CF-3		ND	ND	ND	ND	0.15 g	-	-	-	**-**
YCF1		36.30	ND	37.28	ND	0.16 g	36.30	37.28	**18.79**	113.95
YCF2		36.89	38.58	n.d.	36.22	0.16 g	37.74	36.22	**8.15**	248.69
YCF3		37.30	ND	ND	ND	0.15 g	37.30	-	**11.98**	-
CF2.1		ND	ND	36.38	37.09	0.15 g	-	36.74	-	**330.93**
CF2.2		37.24	ND	36.63	ND	0.15 g	37.24	36.63	**23.52**	355.28
CF2.3		ND	ND	38.67	ND	0.15 g	-	38.67[a]	-	UDL
CF3.1		ND	ND	ND	ND	0.15 g	-	-	-	**-**
CF3.2		ND	ND	ND	ND	0.15 g	-	-	-	**-**
CF3.3		ND	ND	ND	ND	0.15 g	-	-	-	**-**
YC-CM1		36.65	ND	ND	ND	0.15 g	36.65	-	**29.63**	**-**
YC-AM1		ND	ND	ND	36.37	0.15 g	-	36.37	-	**360.01**
TDB-1	Thermophilic digester for treating biowaste	ND	ND	ND	ND	0.20 g	-	-	-	-
TD-1	Thermophilic digester for treating sewage sludge	41.31[b]	35.43	37.47	35.78	0.20g	35.43	36.63	**47.09**	208.93
TD-2		ND	ND	ND	38.25	0.15 g	-	38.25[a]	-	UDL
Soil-1	Soil (collected from cattle farm)	ND	ND	ND	36.55	0.15 g	-	36.55	**-**	**375.63**
Soil-2		37.86	n.d.	37.38	37.64	0.15 g	37.86	37.51	**14.91**	190.83
MMF-1	Mixed storage cow manure	36.85	ND	ND	42.42	0.15 g	36.85	42.42[a]	**16.68**	UDL
MMF-1		36.18	37.16	ND	ND	0.15 g	36.67	-	**19.05**	-

MMO-1		37.25	n.d.	38.58	ND	0.15 g	37.25	38.58[a]	**19.46**	UDL
MMO-2		ND	37	ND	ND	0.15 g	37.00	-	**19.36**	-
SMM-1		36.80	ND	ND	ND	0.22 g	36.80	-	**15.09**	-
SMM2		ND	ND	ND	ND	0.15 g	-	-	-	-
BP1	Biogas plant	ND	ND	ND	ND	0.40 g	-	-	-	-
BP2		ND	ND	ND	ND	0.21 g	-	-	-	-
FFC	Adult cow faeces	ND	ND	ND	ND	0.15 g	-	-	-	-
C1.1		ND	ND	ND	ND	0.15 g	-	-	-	-
C1.2		ND	ND	ND	ND	0.20 g	-	-	-	-
C1.3		ND	ND	ND	ND	0.20 g	-	-	-	-
GS-1	Grass silage	35.82	36.97	37.37	ND	Pellet of 5 g	36.40	37.37	**1.12**	5.71
MS-1	Maize silage	37.52	ND	ND	ND	Pellet of 5 g	37.52	-	**0.49**	-
HF1	Horse faeces	38.20	ND	ND	ND	Pellet of 5 g	38.20[a]	-	UDL	-
HF2	Horse faeces	ND	ND	ND	ND	Pellet of 0.5 g	-	-	-	-
HF3		35.75	ND	ND	ND	0.13 g	35.75	-	**63.93**	-
HF4		ND	ND	ND	ND	0.20 g	-	-	-	-
HF6		ND	ND	ND	ND	0.25 g	-	-	-	-
HF7		ND	ND	ND	ND	0.25 g	-	-	-	-
HF8		37.36	n.d.	38.91	ND	0.16 g	37.36	38.91[a]	**16.33**	UDL
HF9		ND	ND	ND	ND	Pellet of 5 g	-	-	-	-
HF10		ND	ND	ND	ND	0.26 g	-	-	-	-

[a]UDL: under the detection limit of CD 16S rRNA gene. [b]Major difference replicates (discarded results). ND: not determined. Bold values: Final quantification results depend on Cq values.

Table S2. Comparison of detection results of environmental *C. difficile* between qPCR and *C. difficile* selective enrichment culture (CSEC).

Farm No / Sample ID	Sample No.	Sample Type	No. of cases					
			qPCR		CSEC		qPCR and CSEC	
			+	-	+	-	+	-
Farm 1	3	Calf faeces	0	3	1	2	0	2
Farm 2	3	Calf faeces	0	3	0	3	0	3
Farm 3	3	Calf faeces	0	3	0	3	0	3
Farm 4	3	Calf faeces	3	0	3	0	3	0
Farm 5	2	Biogas plant	0	2	2	0	0	0
Farm 6	3	Calf faeces	2	1	3	0	2	0
Farm 7	3	Calf faeces	1	2	3	0	1	0
Farm 8	4	Adult cow faeces	0	4	0	4	0	4
	8	Calf faeces	4	4	0	8	0	4
	2	Mixed storage manure	1	1	0	2	0	1
	1	Grass silage	1	0	0	1	0	0
	1	Maize silage	1	0	0	1	0	0
Farm 9	4	Mixed storage cow manure	4	0	0	4	0	0
	6	Calf faeces	4	2	0	6	0	2
	2	Soil (collected from cattle farm)	2	0	1	1	1	0
WWTP samples	4	Raw sewage (influent)	4	0	4	0	4	0
	3	Treated sewage (effluent)	2	1	0	3	0	1
	4	Raw sewage sludge	4	0	3	1	3	0
	4	Digested sewage sludge	3	1	4	0	3	0
	1	Activated sewage sludge	1	0	1	0	1	0
S-DSD	3	Digested sludge-amended soils	3	0	3	0	3	0
TD/TDB	3	Thermophilic digester for treating sewage sludge or biowaste	1	2	2	1	1	1
Anaerobic lab-scale bioreactors	2	Thermophilic digestion of sewage sludge: Control / Experiment	2	0	2	0	2	0
HF	9	Horse faeces	2	7	0	9	0	7
Total	**81**		**45**	36	**32**	49	**24**	28
